# Beyond the Debye Temperature: A Unified Microscopic Framework for Designing Thermally Stable Red‐Emitting Phosphors

**DOI:** 10.1002/advs.78623

**Published:** 2026-11-09

**Authors:** Noolu. Srinivasa Manikanta Viswanath, Jeong Hui Kim, Yeongjun Yoon, Sung Woo Jang, Sanjith Unithrattil, San Ha Choi, Kyeounghak Kim, Won Bin Im

**Affiliations:** ^1^ Division of Materials Science and Engineering Hanyang University Seoul Republic of Korea; ^2^ Division of Chemical Engineering Hanyang University Seoul Republic of Korea

**Keywords:** color‐conversion photonics, debye temperature, local dipole engineering, symmetry‐breaking phosphors, thermal quenching mechanisms, vibronic renormalization

## Abstract

Symmetry‐breaking luminescent materials enhance light–matter interactions and electric‐dipole transitions, yet the links between local symmetry, dipole modulation, and thermal quenching remain elusive. Two new noncentrosymmetric Eu^3+^‐activated oxide phosphors, Na_3_EuSi_3_O_9_ (NESO) and Na_3_EuGe_3_O_9_ (NEGO), are reported and serve to establish a unified microscopic framework that correlates local symmetry, dipole modulation, and thermal quenching behavior with luminescence performance. Despite sharing the nonpolar P2_1_2_1_2_1_ structure, Ge substitution strengthens EuO_6_ bonding, enhancing local dipoles and light–matter interactions. Consequently, NEGO exhibits a photoluminescence quantum yield of 88%, retains 95% of its emission after three days in water immersion, and retains 91% at 200°C, outperforming commercial K_2_SiF_6_:Mn^4+^ and CaAlSiN_3_:Eu^2+^ phosphors. Combined spectroscopy and DFT reveal that NESO has a stiffer lattice, while NEGO exhibits lower phonon energies, stronger Eu─O bonding, and greater Eu‐centered structural and electronic stability. These findings identify local activator response, rather than bulk lattice rigidity alone, as a key descriptor of thermal robustness. Integrating local symmetry engineering with microscopic insights into thermal quenching provides a design principle for high‐performance rare‐earth phosphors, advancing color conversion, 3D‐printed photonic devices, and solid‐state lighting.

## Graphical abstract

Local symmetry engineering in noncentrosymmetric Na_3_EuGe_3_O_9_ generates enhanced Eu^3+^ local dipoles, enabling efficient light–matter interactions, high quantum efficiency, and exceptional thermal stability. A unified microscopic framework reveals the cooperative suppression of multiple thermal‐quenching pathways, resulting in robust red emission in high‐performance UV‐driven color‐converting devices and complex 3D‐printed photonic architectures.

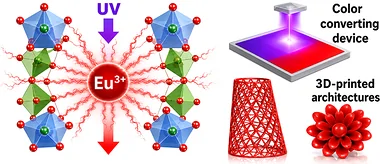

## 1 Introduction

Color‐converting devices are essential for displays, lighting, sensing, and emerging 3D‐printing technologies, where light is converted into desired colors with high efficiency. By reducing energy consumption and improving light utilization, they support SDG 7 (Affordable and Clean Energy), whereas their role in advanced photonic technologies aligns with SDG 9 (Industry, Innovation and Infrastructure) and contributes to SDG 13 (Climate Action) through improved energy efficiency [1, 2, 3]. Stable and efficient red color converters are particularly important because they improve color purity, display performance, and lighting quality, making them key components in next‐generation color‐conversion devices and printable photonic applications [4, 5, 6, 7].

Red‐emitting phosphors are essential for color‐conversion applications, enabling high color rendering, warm white light, and a wide color gamut in solid‐state lighting and displays [8, 9, 10]. Commercial phosphors such as K_2_SiF_6_:Mn^4+^ (KSF) and SrLiAl_3_N_4_:Eu^2+^ offer narrowband red emission, high color purity, and excellent thermal performance; however, their long‐term stability remains a concern [11, 12, 13, 14]. Despite their excellent optical performance, the limitations of current commercial red phosphors highlight the need for new red‐emitting materials that combine high efficiency, narrowband emission, and superior environmental and thermal stability. However, identifying a thermally stable red‐emitting phosphor is challenging because its red emission is highly susceptible to thermally activated non‐radiative relaxation, including multi‐phonon processes, charge‐transfer transitions, and migration to quenching centers. Moreover, red‐emitting activators such as Eu^3+^, Eu^2+^, and Mn^4+^ are sensitive to their local crystal environments, making it difficult to simultaneously achieve high efficiency, narrowband emission, and excellent thermal stability.

The Debye temperature (Θ_D_) is widely used as a descriptor of thermal stability because it reflects lattice stiffness and characteristic phonon energy [15, 16, 17, 18, 19].A higher Θ_D_ generally indicates a stiffer lattice and reduced multiphonon nonradiative relaxation. However, Θ_D_ alone does not always accurately predict thermal quenching. For example, the excellent thermal stability of nitride phosphors, such as CaAlSiN_3_:Eu^2+^ and SrLiAl_3_N_4_:Eu^2+^, is governed not only by lattice rigidity but also by strong metal–nitrogen bonding and activator–host interactions, whereas Y_3_Al_5_O_12_:Ce^3+^ and K_2_SiF_6_:Mn^4+^ can undergo thermal quenching through activator‐specific concentration effects and electronic processes that are not directly captured by the bulk Debye temperature [20, 21, 22]. The limitation of Θ_D_ arises from its nature as a macroscopic, averaged descriptor of the phonon spectrum. It does not directly capture the local activator environment, specific phonon modes, activator–ligand bonding, structural distortions, defects, or energy‐migration pathways. Moreover, thermal quenching may be dominated by electronic processes such as charge transfer, carrier ionization, excited‐state crossover, and configurational‐coordinate displacement, which are not necessarily correlated with lattice stiffness [23, 24]. Thus, a phosphor with a high Θ_D_ may still exhibit severe thermal quenching, whereas a material with a lower Θ_D_ can remain thermally stable when strong local activator–host interactions suppress electronic quenching pathways. Therefore, Θ_D_ should be regarded as a useful first‐order indicator of lattice dynamics rather than a universal predictor of thermal stability. A more reliable assessment of thermal quenching requires integrating bulk lattice descriptors with local activator–host bonding, electronic band alignment, phonon–electron coupling, defect chemistry, and thermally activated charge transfer or ionization processes.

In this study, the thermal quenching mechanism in Eu^3+^‐activated red‐emitting phosphors, Na_3_EuSi_3_O_9_ (NESO) and Na_3_EuGe_3_O_9_ (NEGO), was investigated using a combined experimental and theoretical approach. Although conventional models suggest that rigid lattices with higher Debye temperatures (Θ_D_) should exhibit superior thermal stability, NEGO showed significantly greater resistance to thermal quenching and a higher photoluminescence quantum yield than NESO. To understand this anomalous behavior, detailed analyses of the electronic structure, local chemical bonding, phonons, and Zacharias–Giustino (ZG) special‐displacement configurations were performed [25]. The calculations distinguish bulk lattice stiffness from the local response of the EuO_6_ emitting center. Although NESO has a higher Debye temperature, stronger Si─O framework bonding, and a higher phonon cutoff, NEGO exhibits stronger average Eu─O bonding and smaller displacement‐induced changes in the EuO_6_ geometry and O 2p–Eu 4f level alignment. The latter quantity is used as a qualitative descriptor of charge‐transfer susceptibility rather than as a calculated hole‐ionization barrier. These results associate the enhanced thermal stability of NEGO with lower host phonon energies and reduced local vibronic sensitivity, without assigning a unique dominant nonradiative pathway. As a proof of concept, a red color‐conversion device fabricated using NEGO delivered a high luminance of 2000 cd m^−2^ at 4.2 V. Furthermore, the excellent luminescence and processability of NEGO were demonstrated through the successful fabrication of three‐dimensional octahedral and cubic Platonic solid architectures via 3D printing, highlighting its potential for next‐generation color‐conversion devices and additive‐manufactured photonic applications.

## 2 Results and Discussion

Na_3_EuM_3_O_9_ (M = Si, Ge) crystallizes in the non‐centrosymmetric orthorhombic space group *P2_1_2_1_2_1,_* forming a three‐dimensional framework of corner‐sharing MO_4_ (M = Si, Ge) tetrahedra interconnected by oxygen atoms, with Eu^3+^ ions fully occupying the rare‐earth sites within the lattice (Figure 1a). Unlike conventional activator‐doped phosphors, Eu^3+^ ions are an intrinsic constituent of the host structure, resulting in a dense and rigid Eu─O network. Notably, Eu^3+^‐centered polyhedra are arranged in a one‐dimensional helical arrangement, generating unique structural connectivity that influences the local symmetry, energy transfer dynamics, and lattice stability [26, 27, 28]. The non‐centrosymmetric helical framework enhances the asymmetry around the Eu^3+^ ions, strengthening the electric‐dipole‐allowed ^5^D_0_→^7^F_2_ transition responsible for intense red emission [29]. The structure contains four crystallographically distinct Eu sites (Eu1–Eu4) with the shortest Eu─Eu separations of 6.59, 6.92, 7.67, and 8.98 Å, respectively (Figure 1b). These relatively large Eu─Eu distances effectively isolate neighboring Eu^3+^ centers, minimizing direct exchange interactions and suppressing nonradiative energy migration despite complete Eu^3+^ ion occupation. Furthermore, the directional connectivity of the spiral Eu network regulates excitation energy transfer pathways, whereas the three‐dimensional framework suppresses lattice vibrations and concentration quenching, restricts thermally activated carrier loss, and stabilizes the excited states, thereby promoting highly efficient and robust red emission suitable for photonic applications. To further elucidate the local electronic environment, band structure and density of states (DOS) calculations were performed using spin‐polarized density functional theory (DFT) for both Na_3_EuSi_3_O_9_ (NESO) and Na_3_EuGe_3_O_9_ (NEGO) [30]. As shown in Figures S1 and S2, both compounds exhibit spin‐polarized electronic structures with wide bandgap, confirming their highly insulating nature. NESO exhibits a direct bandgap, whereas NEGO displays a quasi‐direct bandgap with nearly identical direct and indirect bandgap energies. The valence band maximum is located near the Fermi level, whereas the conduction band minima appear at higher energies, minimizing host‐lattice absorption and suggesting suppression of nonradiative recombination. In both materials, dispersionless bands are observed at approximately 3.3–3.6 eV above the Fermi level in the spin‐up channel, originating from highly localized Eu 4*f* states. These results agree with UV–vis's absorption measurements, which yield optical bandgaps of 3.4 eV for NESO and 3.1 eV for NEGO, confirming the wider bandgap of the silicate analogue (Figure S3). The pronounced spin splitting between the spin‐up and spin‐down channels arises from the partially filled Eu 4*f* shell, indicating magnetic polarization of the electronic structure. This interpretation is further supported by the spin‐polarized DOS profiles (Figure 1c,d), in which the valence band is dominated by O 2p states, whereas the conduction band primarily consists of Si/Ge‐derived states with contributions from Eu 4*f* orbitals. Distinct localized Eu 4*f* states are clearly observed within the bandgap region, confirming the strong localization of Eu electrons and their key role in the luminescence process. The unique combination of localized Eu 4*f* states, wide bandgaps, and a rigid helical Eu─O network is expected to promote efficient radiative recombination while suppressing carrier trapping and nonradiative losses. These attributes make NESO and NEGO attractive candidates for thermally robust, highly efficient red‐emitting materials, motivating their synthesis and detailed investigation. NESO and NEGO were synthesized via a conventional high‐temperature solid‐state reaction route, and their experimentally determined properties were subsequently compared with the calculated structural and electronic descriptors (experimental details are provided in the Supporting Information). The phase purity and crystal structures of the as‐synthesized samples were examined by powder X‐ray diffraction (XRD), followed by Rietveld refinement using the GSAS package (Figure 1e) [31]. The excellent agreement between the observed and calculated diffraction patterns confirmed the successful formation of phase‐pure NESO and NEGO, with no detectable secondary phases.

**FIGURE 1:**
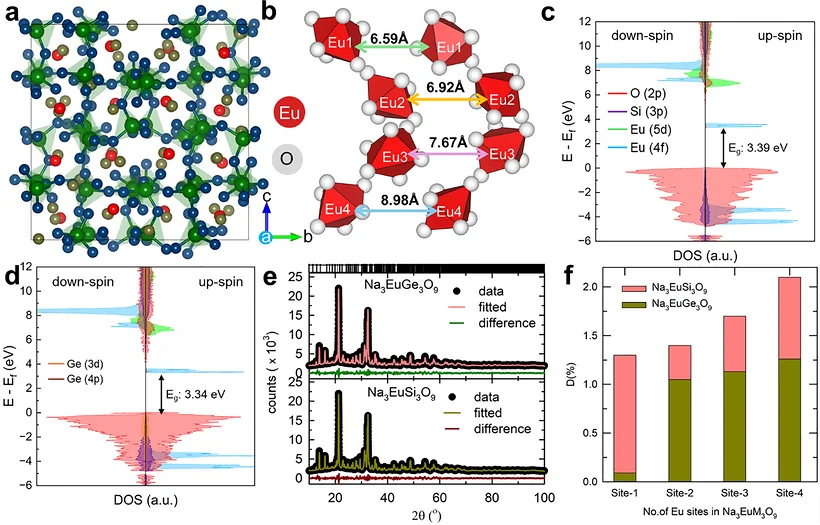
Local structure and electronic structure properties of red emitting phosphors (a) Crystal structure of Na_3_EuM_3_O_9_ (M = Si, Ge) crystallizing in the non‐centrosymmetric orthorhombic space group *P2_1_2_1_2_1_*, showing the three‐dimensional framework of corner shared [MO_4_] tetrahedra and Eu‐centered polyhedra. (b) Schematic illustration of the helical Eu─O network highlighting the four crystallographically distinct Eu sites (Eu1–Eu4) and the shortest Eu─Eu separations. Spin‐polarized total and partial DOS of (c) NESO and (d) NEGO, showing the contributions of Eu, O, and Si/Ge orbitals to the electronic structure. (e) Rietveld refinement profiles of the powder x‐ray diffraction patterns for NEGO and NESO, confirming the formation of phase‐pure compounds. Black circles and solid lines represent the experimental and calculated patterns, respectively, while the tick marks indicate Bragg reflection positions, and green and red line represents the difference profile after full pattern refinement. (f) Distortion indices (D) of the four crystallographically distinct Eu sites in NESO and NEGO, revealing site‐dependent differences in the local Eu coordination geometry.

The refined structural parameters and reliability factors are summarized in Tables S1–S3. After full refinement convergence, the distortion indices of the crystallographically distinct Eu sites were extracted to evaluate the local structural environment (Figure 1f). The analysis revealed that NEGO provides a more robust and strongly bonded local coordination environment around the Eu^3+^ ions than NESO, which is expected to influence the local crystal field, suppress localized thermal distortions, and enhance resistance to thermal quenching, thereby affecting the luminescence performance of the materials. Scanning electron microscopy (SEM) was used to examine the morphology of the synthesized samples. Both NESO and NEGO exhibit well‐developed particles with sizes ranging from approximately 250 to 300 µm, indicating substantial crystal growth during high‐temperature solid‐state synthesis (Figure S4). The formation of these large particles resembling single‐crystal‐like grains can be attributed to the high crystallinity and prolonged diffusion‐driven grain growth facilitated by the elevated reaction temperature [32, 33]. The dense, rigid crystal framework may further promote crystal coarsening, while minimizing the formation of grain boundaries. Elemental mapping analysis confirmed the homogeneous distribution of Na, Eu, Si/Ge, and O throughout the crystal, demonstrating the successful incorporation of all constituent elements without detectable phase segregation or compositional inhomogeneity (Figures S5 and S6). This uniform elemental distribution, together with the large crystal domains, further supports the high phase purity and structural integrity of the synthesized materials.

To investigate the luminescence properties of NESO and NEGO, the samples were excited with 400 nm light, and their photoluminescence excitation (PLE) and emission (PL) spectra were measured (Figure 2a,b). The PLE spectra, monitored at 610–612 nm, show the characteristic intraconfigurational Eu^3+^ excitation transitions, while the PL spectra obtained under 400 nm excitation display the typical ^5^D_0_→^7^F_J_ (J = 1–4) emissions. The dominant ^5^D_0_→^7^F_2_ transition at ∼610–612 nm confirms that Eu^3+^ occupies a non‐centrosymmetric site in both hosts [34]. Compared with NESO, NEGO exhibits a noticeably stronger direct excitation band in the 500–550 nm region, which can be attributed to Ge‐induced local structural distortion, enhanced Eu─O covalency, and stronger crystal‐field interactions that further break the local symmetry around Eu^3^^+^ and relax the parity selection rules [35]. These structural perturbations increase the oscillator strength of the electric‐dipole‐allowed *4f*–*4f* transitions, enabling more efficient population of the emitting ^5^D_0_ state [36]. This enhanced local symmetry is further supported by the Eu^3^^+^ asymmetry ratio (R = I^5^D_0_→^7^F_2_/I^5^D_0_→^7^F_1_), which increases from approximately 2.1 for NESO to approximately 5.0 for NEGO, indicating a substantially more asymmetric local coordination environment around the Eu^3^^+^ ions in NEGO. To quantitatively determine how these structural changes, affect the electronic transition probabilities, Judd–Ofelt analysis was performed, as it provides quantitative intensity parameters that directly describe the effects of local symmetry, covalency, and crystal‐field interactions on rare‐earth *4f*–*4f* transitions. Accordingly, the Judd–Ofelt intensity parameter Ω_2_ increases from approximately 5.4 × 10^−^^20^ cm^2^ for NESO to 10.2 × 10^−^^20^ cm^2^ for NEGO, reflecting enhanced Eu─O covalency, stronger local crystal‐field asymmetry, and a larger induced local dipole. The branching ratio of the electric‐dipole‐allowed ^5^D_0_→^7^F_2_ transition also increases from ∼61% to ∼75%, confirming the enhanced electric‐dipole transition probability in NEGO. Collectively, these results establish a direct structure–dipole–luminescence relationship, indicating that Ge‐induced local symmetry modulation enhances the locally induced dipole and electric‐dipole transition probabilities, thereby contributing to the superior luminescence performance of NEGO compared with NESO [37, 38]. To quantitatively assess the resulting enhancement in emission efficiency, the absolute photoluminescence quantum yield (PLQY) was measured (Figure 2c). NEGO exhibits a remarkably high PLQY of 88%, which substantially exceeds that of NESO (57%). This pronounced enhancement in PLQY can be attributed to the synergistic effects of the larger absorption cross‐section and reduced nonradiative losses in NEGO, enabling a greater fraction of absorbed photons to be converted into radiative emission. In addition, PL lifetime measurements further corroborate these results, with NEGO exhibiting a longer average lifetime of 1.56 ms compared with 1.06 ms for NESO (Figure 2d). The decay profiles were fitted using a biexponential model: 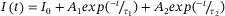 where *I*, and *I*_0_ represent the intensity at time t and 0; *t* is time (s); *A_1_*, and *A_2_* are constants, and *τ_1_*, and *τ_2_*, are the decay times, respectively. The fitted residual parameters are shown in the Table S4. The prolonged emission lifetime of NEGO indicates more efficient radiative recombination and suppressed nonradiative relaxation, which is in excellent agreement with its significantly higher PLQY. The radiative and nonradiative decay rates were quantitatively evaluated to clarify the origin of the enhanced luminescence efficiency. The estimated radiative lifetimes are 1.88 and 1.76 ms, corresponding to radiative transition rates of 533 and 567 s^−^^1^ for NESO and NEGO, respectively. Notably, the nonradiative decay rate decreases dramatically from 402 s^−^^1^ for NESO to only 70 s^−^^1^ for NEGO, representing an approximately six‐fold suppression of nonradiative relaxation. To further understand the origin of the superior PLQY in NEGO, the spatial distribution of Eu^3+^ ions was analyzed, as shown in Figure 2e,f. Although both compounds share the same crystal structure, NEGO exhibits a slightly larger average Eu─Eu separation (6.59–6.92 Å) than NESO (6.51–6.65 Å). The increased Eu─Eu distance weakens resonant energy transfer between neighboring Eu^3+^ ions, thereby suppressing concentration quenching and cross‐relaxation processes that normally serve as nonradiative decay pathways [39, 40, 41]. As a result, the excitation energy remains localized on the emitting Eu^3+^ centers and is more efficiently released through radiative transitions. These results demonstrate that the superior luminescence efficiency of NEGO originates primarily from the effective suppression of nonradiative decay rather than from a significant enhancement of the intrinsic radiative transition rate, whereas its enhanced absorption and electric‐dipole transition probability further facilitate efficient photon conversion.

**FIGURE 2:**
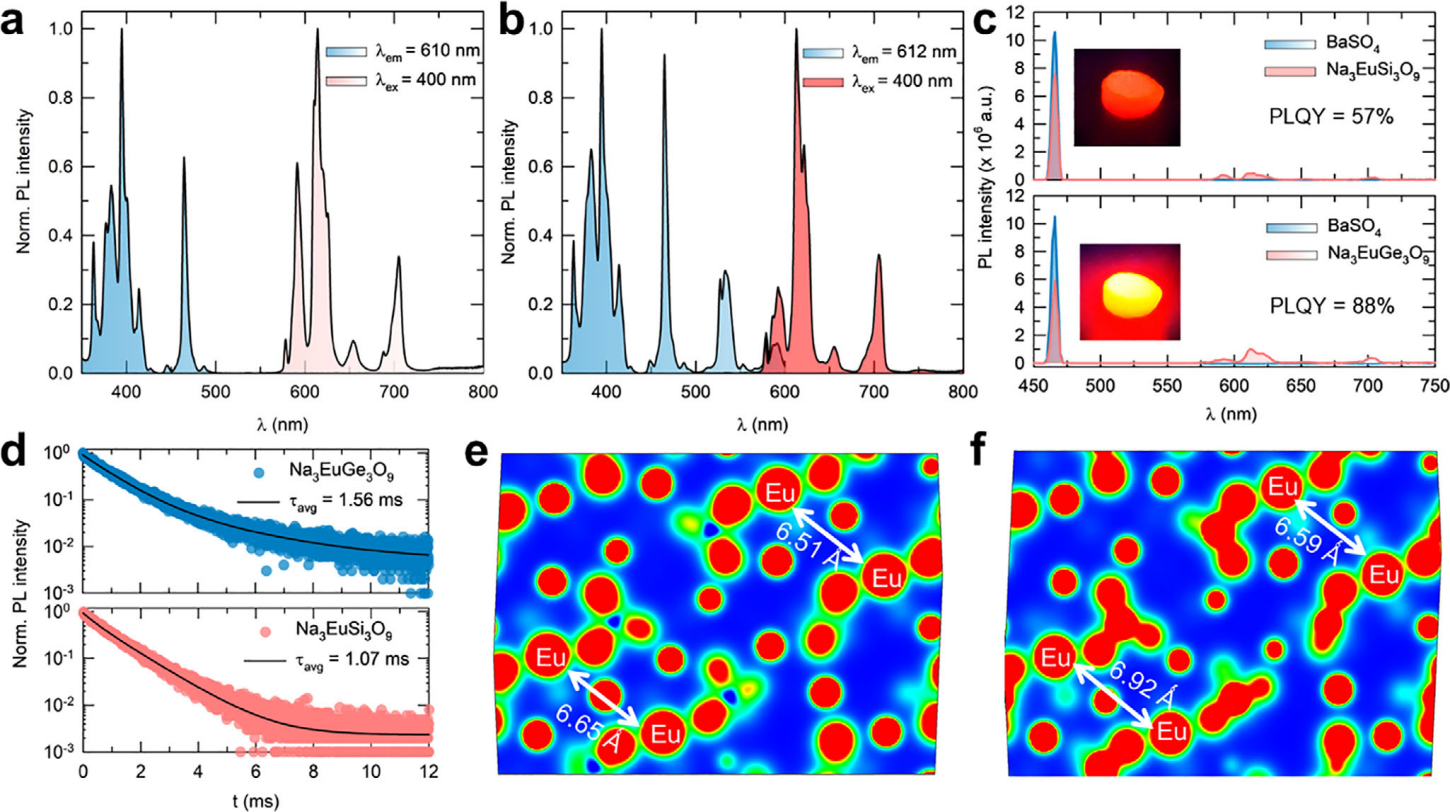
Photophysical properties and the origin of the enhanced luminescence in NESO and NEGO. (a,b) Excitation (λ_e__m_ = 610 and 612 nm) and emission (λ_e__x_ = 400 nm) spectra of NESO and NEGO, respectively, showing the characteristic Eu^3+^ transitions and the enhanced excitation efficiency in NEGO. (c) Absolute PLQY measurements using BaSO_4_ as a reference, revealing a significantly higher PLQY of 88% for NEGO compared with 57% for NESO, accompanied by photographs of the corresponding red emissions under 465 nm excitation. (d) Time‐resolved PL decay profiles showing a longer average lifetime for NEGO (τ_a__v__g_ = 1.56 ms) than that for NESO (τ_a__v__g_ = 1.07 ms), indicating reduced nonradiative recombination and enhanced radiative efficiency. (e,f) Spatial distribution maps of Eu^3+^ ions illustrating the nearest‐neighbor Eu─Eu separations in NESO and NEGO, respectively.

To elucidate the thermal quenching behavior of NESO and NEGO, temperature‐dependent PL spectra were recorded from 298 to 473K, as shown in Figure 3a,b. Both phosphors exhibit the characteristic Eu^3+^ emissions originating from the ^5^D_0_→^7^F_J_ (J = 1–4) transitions, with the dominant red emission centered at approximately 610–612 nm. As the temperature increases, the PL intensity of both phosphors gradually decreases because of thermally activated nonradiative relaxation processes [42, 43, 44, 45]. However, the intensity attenuation is significantly smaller for NEGO than for NESO, demonstrating the superior thermal‐quenching resistance of NEGO. Notably, the emission profiles and peak positions remain essentially unchanged over the entire temperature range, indicating that the Eu^3+^ luminescence centers are structurally stable and that the reduction in emission intensity arises primarily from thermally activated nonradiative processes rather than any phase transition, unlike that observed in the Na_3_Sc_2_(PO_4_)_3_: Eu^2+^ phosphor [46]. Figure 3c compares the normalized PL intensities of NESO and NEGO with those of the commercial red phosphor KSF:Mn^4+^ and the commercial orange‐red phosphor CaAlSiN_3_:Eu^2+^. At 473K, NEGO retains approximately 91% of its initial room‐temperature emission intensity, which is substantially higher than that of NESO (74%) and also exceeds the thermal stability of commercial KSF: Mn^4+^ (79%) and CaAlSiN_3_:Eu^2+^ (88%). The thermal stability reliability of NEGO was further evaluated through temperature‐dependent PL measurements and repeated heating–cooling cycles. As shown in Figure S7a, the PL intensity remained nearly unchanged up to 373K and retained approximately 92% of its initial intensity at 473K. More importantly, the PL intensity recovered reproducibly upon cooling, indicating that the thermally induced PL attenuation is largely reversible without significant permanent degradation. Repeated heating–cooling measurements over six cycles (Figure S7b) exhibited highly reproducible PL responses, with no progressive loss of emission intensity. In addition, the emission spectra recorded before and after thermal treatment (Figure S7(c–h)) show nearly identical spectral profiles and peak positions, confirming the preservation of the Eu^3^^+^ emission characteristics. These results collectively demonstrate the excellent short‐term thermal robustness and reversibility of NEGO up to 473K. The enhanced TQ resistance of NEGO was further quantified using Arrhenius analysis. As shown in Figure S8, the plots of ln((I_0_/I_T_)−1) vs. 1000/T exhibit good linearity, yielding activation energies of 0.141 eV for NESO and 0.246 eV for NEGO. The activation barrier of NEGO is therefore approximately 1.74 times higher than that of NESO, indicating that a substantially larger thermal energy is required to activate the quenching pathway in NEGO. This higher activation barrier suppresses thermally activated non‐radiative relaxation and facilitates the retention of Eu^3^^+^ emission at elevated temperatures.

**FIGURE 3:**
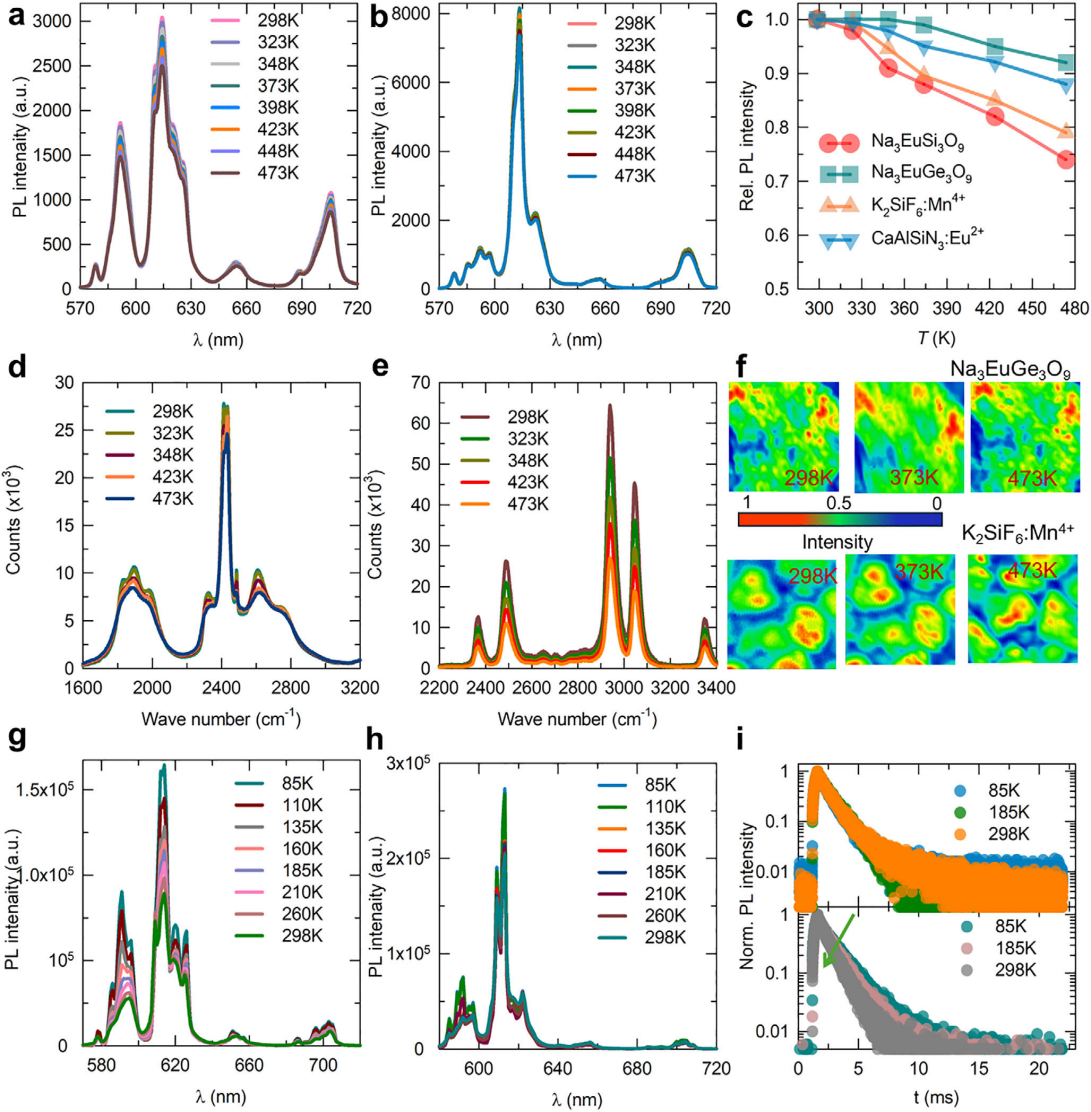
Temperature‐dependent PL, Raman spectroscopy, Raman mapping, and PL decay characteristics of NESO and NEGO phosphors. (a,b) Temperature‐dependent PL spectra of NESO and NEGO, respectively, recorded over the temperature range of 298K–473K under identical excitation conditions. (c) Relative PL intensity as a function of temperature for NESO and NEGO, together with the commercial red phosphor KSF: Mn^4+^ and the commercial orange‐red phosphor CaAlSiN_3_:Eu^2+^ for comparison. (d,e) Temperature‐dependent Raman spectra of NEGO and KSF: Mn^4+^, respectively, measured from 298 to 473K, displaying the evolution of the characteristic Raman bands with increasing temperature. (f) Raman mapping images of NEGO and KSF: Mn^4+^ collected at 25°C, 100°C, and 200°C by monitoring the characteristic Raman bands centered at approximately 2400 cm^−1^ (NEGO) and 2900 cm^−1^ (KSF), illustrating the spatial distribution of the respective phosphors at different temperatures. (g,h) Low‐temperature PL spectra of NESO and NEGO recorded from 85 to 298 K, exhibiting the temperature‐dependent evolution of the Eu^3+^emission bands. (i) Time‐resolved PL decay curves of NEGO (top) and NESO (bottom) measured at 85, 185, and 298 K, showing the emission decay profiles as a function of temperature.

The aqueous stabilities of the KSF: Mn^4+^ and NEGO phosphors were evaluated by immersing the samples in water. After 3 days, NEGO retained approximately 95% of its initial PL intensity, demonstrating excellent water stability, whereas the KSF: Mn^4+^ phosphor exhibited severe degradation, retaining only approximately 5% of its original PL intensity (Figure S9a,b). This remarkable difference is attributed to the distinct crystal structures and chemical stabilities of the host lattices. The robust germanate framework of NEGO, composed of strongly bonded Ge─O polyhedra, provides a rigid, hydrophobic environment that protects the Eu^3+^ emission centers from water‐induced degradation. In contrast, KSF: Mn^4+^phosphors containing a fluoride‐based host lattice are highly susceptible to hydrolysis and surface dissolution in aqueous environments, leading to structural degradation and loss of Mn^4+^ luminescence. To further evaluate the water stability and ensure the reliability and reproducibility of the results, four independent samples were synthesized and subjected to water immersion tests. XRD, SEM, and PL measurements were performed before and after water immersion. As shown in Figure S10a, the XRD patterns before and after immersion exhibit nearly identical characteristic diffraction peaks, with no noticeable appearance or disappearance of peaks, confirming that the crystal structure remains stable in water. The SEM images in Figure S10b show that although the morphology becomes somewhat more dispersed after immersion, the overall structural framework is retained without significant degradation. Furthermore, as shown in Figure S10c, the PL spectra of the four independently prepared samples after 3 days of water immersion show highly consistent emission profiles and peak positions, particularly the strong emission around 615 nm, demonstrating excellent reproducibility of the optical response. The consistent PL behavior among the four samples, together with the preserved crystal structure and morphology, confirms the reliable water stability and good reproducibility of the material under aqueous conditions.

To further understand the origin of this exceptional thermal stability, temperature‐dependent Raman spectroscopy and Raman mapping were performed on NEGO and KSF: Mn^4+^ from 298 to 473K (Figure 3d,e). The Raman spectra exhibited several characteristic high‐wavenumber bands corresponding to higher‐order (overtone and combination) phonon modes associated with the GeO_4_ tetrahedral framework in NEGO and the SiF_6_ octahedral framework in KSF: Mn^4+^ [47, 48]. As the temperature increases, the Raman intensities of both phosphors gradually decrease because of the enhanced phonon populations and lattice anharmonicity. However, the reduction is considerably smaller for NEGO than for KSF: Mn^4+^, indicating that the GeO_4_ framework is significantly more rigid and less susceptible to thermally induced lattice distortion. Raman mapping, performed by monitoring the characteristic bands at approximately 2400 cm^−1^ for NEGO and 2900 cm^−1^ for KSF: Mn^4+^, further confirms that both phosphors maintain their spatial distribution upon heating, while NEGO consistently preserves stronger Raman contrast and higher structural uniformity throughout the investigated temperature range (Figure 3f). These findings demonstrate that the rigid GeO_4_ framework effectively suppresses lattice vibrations and phonon‐assisted structural disorder, thereby stabilizing the local Eu^3+^ coordination environment and reducing nonradiative relaxation.

Nevertheless, although NESO and NEGO crystallize in the same noncentrosymmetric structure and contain the same Eu^3^^+^ luminescent center, they exhibit markedly different thermal quenching behaviors. To elucidate the origin of this difference, temperature‐dependent PL spectra at low temperatures and decay dynamics were investigated over the range of 85–298 K, as shown in Figure 3g–i. As the temperature increases, the emission intensity of both phosphors gradually decreases because of thermally activated nonradiative relaxation. However, NEGO exhibits a substantially smaller reduction in PL intensity than NESO, indicating significantly greater resistance to thermal quenching. Consistently, the temperature‐dependent decay curves (Figure 3i) show that the emission lifetime of NEGO decreases only slightly with increasing temperature, whereas NESO undergoes pronounced lifetime shortening, demonstrating that nonradiative recombination channels are much more effectively suppressed in NEGO. This superior thermal stability cannot be explained solely by bulk lattice rigidity and is therefore examined below in terms of local Eu─O bonding, host phonon energies, and the displacement sensitivity of the Eu‐centered environment. DFT calculations were performed to gain a deeper microscopic understanding of the distinct thermal‐quenching behaviors of NESO and NEGO. Although both phosphors crystallize in the same orthorhombic structure and contain identical Eu^3+^ luminescent centers, they exhibit markedly different thermal stabilities, indicating that thermal quenching is governed by multiple competing nonradiative processes rather than by a single structural descriptor. In general, thermal quenching of Eu^3+^ luminescence can originate from several microscopic mechanisms, including (i) thermally activated charge‐transfer (CT) or hole‐ionization between the Eu^3+^ excited state and the host valence band, (ii) multi‐phonon relaxation assisted by lattice vibrations, (iii) configurational‐coordinate crossover induced by local structural distortions around the Eu^3+^ activator, (iv) vibronic renormalization arising from electron–phonon coupling, (v) migration‐assisted quenching through resonant energy transfer between neighboring Eu^3^^+^ ions, and (vi) variations in the local chemical bonding environment that determine the stability of the EuO_6_ emitting center. Previous studies by Dorenbos showed that the thermal quenching of Eu^3+^ 4*f*–4*f* emission proceeds predominantly through a CT pathway involving thermally activated hole transfer to the valence band rather than electron excitation into the conduction band [23]. More recently, theoretical models have emphasized that configurational‐coordinate crossover, thermal ionization, and local activator stability collectively govern thermal quenching, suggesting that the local bonding environment is a more reliable predictor than conventional global descriptors such as the Debye temperature (Θ_D_) [49]. Motivated by these concepts, the major thermal‐quenching pathways were investigated by evaluating the Eu 4*f*–valence‐band maximum (VBM) energy separation as a descriptor for CT/hole ionization, the host phonon spectrum for multi‐phonon relaxation, the EuO_6_ distortion index for configurational‐coordinate crossover, crystal orbital Hamilton population (COHP) analysis for local Eu─O bonding stability [50], Eu─Eu separation for migration‐assisted quenching, and electron–phonon coupling using the Zacharias–Giustino (ZG) special‐displacement method [25]. These descriptors were first evaluated within the equilibrium static lattice and subsequently re‐examined after explicitly incorporating vibronic effects, enabling a comprehensive microscopic understanding of the mechanisms responsible for the markedly different thermal quenching behaviors of the two phosphors.

Following this framework, the CT/hole ionization pathway was first examined using static lattice electronic descriptors. As previously discussed for Figure 1c,d, the projected density of states (pDOS) reveals that the unoccupied Eu 4*f* states lie within the bandgap between the host VBM and the conduction band minimum (CBM). Accordingly, the Eu 4*f–*VBM energy separation serves as a key descriptor for evaluating the accessibility of thermally activated CT/hole‐ionization quenching [23]. Static‐lattice calculations show that this energy separation is slightly larger for the NESO (3.079 eV) than for the NEGO (3.024 eV), suggesting that NESO should exhibit greater resistance to hole ionization and enhanced thermal stability. Consistent with this prediction, the Debye temperature (Θ_D_), obtained from the DFT‐calculated heat capacities (Figure 4a), is also considerably higher for the NESO (417.4 K) than for the NEGO (339.6 K), indicating a more rigid lattice. Together with the slightly smaller optical bandgap of the NEGO, these conventional static‐lattice descriptors would incorrectly predict stronger thermal quenching in NEGO. This contradiction with the experimental observations shows that static electronic descriptors alone are insufficient and that local bonding and temperature‐dependent vibronic effects must be explicitly considered.

**FIGURE 4:**
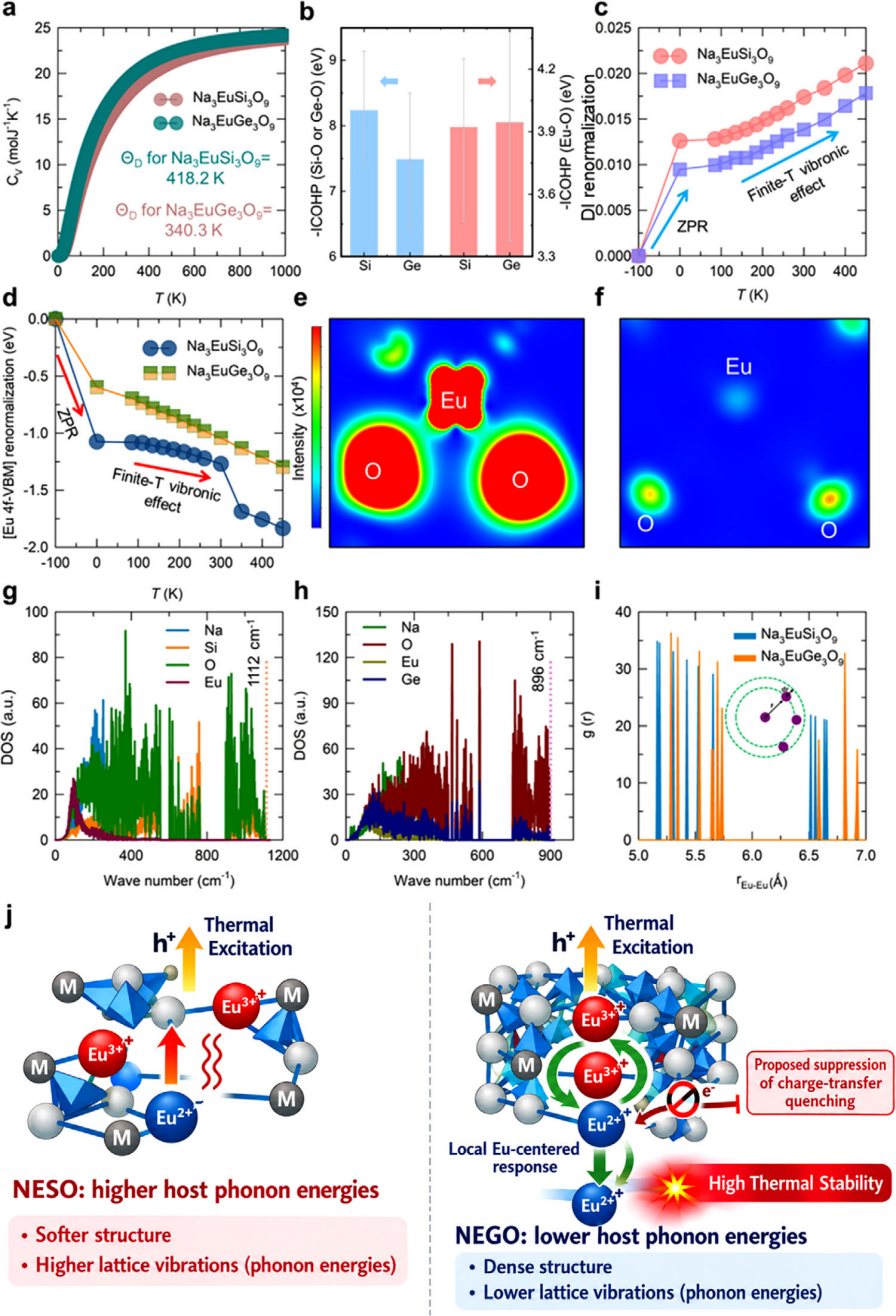
DFT analysis of the microscopic descriptors governing thermal‐quenching behavior in Na_3_EuSi_3_O_9_ and Na_3_EuGe_3_O_9_. (a) DFT‐calculated constant‐volume heat capacity (C_v_) as a function of temperature, together with the corresponding Θ_D_. (b) ICOHP) values comparing the framework bonds (Si─O/Ge─O) and Eu─O coordination, illustrating the relative bond strengths in the two hosts. (c) Temperature‐dependent change in the EuO_6_ polyhedral DI relative to each material's static structure, DI(T) − DI(static), obtained using the ZG special‐displacement approach, including zero‐point and finite‐temperature contributions. (d) Temperature evolution of the Eu 4*f*–VBM energy separation relative to the static lattice, illustrating vibronic renormalization of the CT descriptor. (e,f) Partial charge density distributions corresponding to the valence‐band edge and the unoccupied Eu 4*f* states, respectively, used to evaluate the electronic coupling associated with thermally activated hole transfer. (g,h) Atom‐projected phonon DOS of NESO and NEGO, highlighting the contributions of individual atomic species and the corresponding maximum phonon cutoff frequencies. (i) Eu─Eu RDFs extracted from the structural models, illustrating the nearest‐neighbor Eu─Eu separations relevant to migration‐assisted energy transfer. (j) Proposed mechanism for the enhanced thermal stability of NESO and NEGO. Comparison of host phonon energies and their influence on Eu‐centered CT quenching, highlighting the localized Eu response and suppressed quenching in NEGO.

To understand the origin of this discrepancy, the local chemical bonding environment was examined using ‐ICOHP analysis. ‐ICOHP provides an energy‐resolved measure of bonding interactions, where a larger ‐ICOHP value indicates stronger chemical bonding [51]. As summarized in Figure 4b and Table S6, the Ge─O framework bonds are substantially weaker than the Si─O bonds, with the average ‐ICOHP decreasing from 8.239 to 7.492 eV. In contrast, the Eu─O interaction becomes slightly stronger in the Ge host, increasing from 3.923 to 3.946 eV. These opposite trends indicate a bonding competition (inductive‐like) effect, whereby the weakening of the Ge─O framework redistributes the oxygen electronic density toward the Eu─O bonds, producing a more strongly bonded EuO_6_ coordination environment within a comparatively softer lattice. This local reinforcement is expected to suppress configurational‐coordinate distortions and reduce crossover‐type nonradiative relaxation, thereby establishing a direct relationship between local chemical bonding and thermal robustness [49].

Because thermal quenching is inherently temperature‐dependent, the role of electron–phonon coupling was further investigated by incorporating vibronic effects using the Zacharias–Giustino (ZG) special‐displacement method. This approach captures both quantum zero‐point vibrations and finite‐temperature phonon populations through a representative thermally displaced configuration. The resulting structural response was quantified using the EuO_6_ DI (Figure 4c and Table S7). The static‐lattice DI is higher for NEGO (0.0127) than for NESO (0.0098). We therefore compare the displacement‐induced change relative to each material's static structure, defined as ΔDI(T) = DI(T) − DI(static). At 0 K including zero‐point motion, the DI values are 0.0224 for NESO and 0.0221 for NEGO, corresponding to increases of approximately 0.0126 and 0.0094, respectively. The static‐referenced increase remains smaller in NEGO throughout the calculated 0–450 K range. Thus, the relevant distinction is the smaller displacement‐induced change in the EuO_6_ geometry of NEGO, rather than a lower absolute static distortion. The corresponding electronic response was also examined. Although the static Eu 4*f*–VBM separation is initially larger in the NESO, zero‐point renormalization reduces this gap by 1.074 eV compared with only 0.596 eV for the NEGO. As a result, the Eu 4*f*–VBM separation decreases to 2.005 eV in NESO but remains significantly larger at 2.428 eV in NEGO (Figure 4d). This inversion persists after finite‐temperature vibronic renormalization, with NEGO maintaining a larger Eu 4f–VBM separation (Figure 4d). This trend is consistent with the experimentally observed higher thermal‐quenching resistance of NEGO.

The spatial characteristics of the electronic states associated with the proposed CT pathway were further examined using partial charge density analysis. According to Fermi's golden rule, the transfer probability depends not only on the activation barrier but also on the square of the electronic coupling matrix element [52]. A partial charge density analysis (Figure 4e,f) reveals stronger orbital overlap between the O 2p‐derived valence band states and the Eu‐centered acceptor states in the Si host, whereas the Ge host exhibits a noticeably weaker Eu─O bridging density. The weaker Eu─O bridging density in NEGO suggests reduced spatial mixing between the relevant states, consistent with its lower susceptibility to the proposed charge‐transfer quenching pathway.

Beyond carrier‐transfer processes, the multi‐phonon relaxation pathway was evaluated using the atom‐projected phonon density of states (Figure 4g,h). Owing to the lighter reduced mass and stronger Si–O bonds, the NESO exhibits a substantially higher maximum phonon cutoff frequency (1,112 cm^−1^) than the NEGO (896 cm^−1^). Using the representative Eu^3+^ emission energy gap (ΔE ≈ 12 000 cm^−^^1^) [53], the required phonon numbers are estimated to be approximately 10.8 for the Si host and 13.4 for the Ge host. According to the energy‐gap law [54, 55, 56], the multi‐phonon decay rate decreases exponentially with increasing phonon number. Consequently, the lower phonon energies of the Ge framework intrinsically suppress multi‐phonon relaxation, which is consistent with the experimentally observed temperature‐dependent PL decay, in which NEGO maintains a significantly more stable emission lifetime upon heating.

Finally, migration‐assisted quenching was examined through the spatial distribution of Eu^3+^ ions. Because resonant energy transfer strongly depends on The activator–activator separation, Eu─Eu radial distribution functions (RDFs) were calculated (Figure 4i). The nearest‐neighbor Eu─Eu distance was noticeably larger in the NEGO, indicating greater spatial isolation of the EuO_6_ polyhedra. This increased separation weakens the Eu─Eu electronic coupling, suppresses migration‐assisted energy transfer to quenching centers, and reduces nonradiative energy losses [57, 58].

Based on the phonon and ZG analyses, the contrasting thermal‐quenching behaviors should be discussed by distinguishing global framework properties from the local Eu‐centered response. NESO has the higher Debye temperature and phonon cutoff, whereas NEGO exhibits slightly stronger average Eu─O bonding and a smaller displacement‐induced increase in the EuO_6_ DI relative to its own static structure. The latter does not imply that NEGO has a lower static distortion or a denser lattice. As illustrated in Figure 4j, thermally activated hole transfer between Eu^3+^‐associated states and the host valence band provides a possible nonradiative charge‐transfer pathway. The larger Eu 4f–VBM separation maintained by NEGO after vibronic renormalization, together with its local bonding and static‐referenced DI trends, is consistent with reduced susceptibility to this proposed pathway. Overall, the comparison links the enhanced thermal‐quenching resistance of NEGO to the combined local bonding, displacement‐induced structural and electronic responses, and lower host phonon energies, rather than to greater structural density or lower absolute distortion.

These results collectively demonstrate that the superior thermal stability of NEGO arises not from a single descriptor but from the cooperative suppression of all major thermal‐quenching pathways, including charge transfer, configurational‐coordinate crossover, vibronic renormalization, multi‐phonon relaxation, and migration‐assisted quenching. They also establish a unified microscopic framework for thermal quenching in phosphors by integrating charge transfer, configurational‐coordinate crossover, vibronic renormalization, multi‐phonon relaxation, migration‐assisted quenching, and local bonding effects. Beyond explaining the present system, this framework provides a general mechanistic foundation for understanding thermal quenching and guiding the rational design of high‐performance phosphors with superior luminescence efficiency and thermal robustness.

To demonstrate the practical applicability of NEGO as a high‐performance red color‐conversion phosphor, a proof‐of‐concept color‐converting red‐emitting device was fabricated by coating the phosphor onto a commercial UV LED chip. As shown in Figure 5a, the electroluminescence (EL) spectra recorded under driving voltages of 3.0–4.5 V exhibit the characteristic Eu^3+^ red emission dominated by the ^5^D_0_ → ^7^F_2_ transition at ∼612 nm, with negligible spectral variation as the operating voltage increases, demonstrating excellent voltage‐dependent spectral stability. Moreover, the EL spectrum closely matches the PL spectrum in both peak positions and spectral profiles (Figure S11), indicating that electrical excitation preserves the intrinsic Eu^3+^ emission characteristics without introducing defect‐related or parasitic emissions. The corresponding current–voltage–luminance characteristics (Figure 5b) reveal efficient light conversion, with the luminance rising rapidly after the turn‐on voltage and reaching a maximum of approximately 2.0 × 10^3 ^cd m^−2^ near 4.2 V. Consistently, the Commission Internationale de l'Éclairage (CIE) chromaticity coordinates (Figure 5c) remain nearly unchanged over the applied voltage range, confirming excellent color stability. Furthermore, the fabricated device exhibits good operational stability under continuous operation, retaining 50% of its initial luminance (L_0_ = 1000 cd m^−2^) after approximately 153 min (T_50_) (Figure 5d).

**FIGURE 5:**
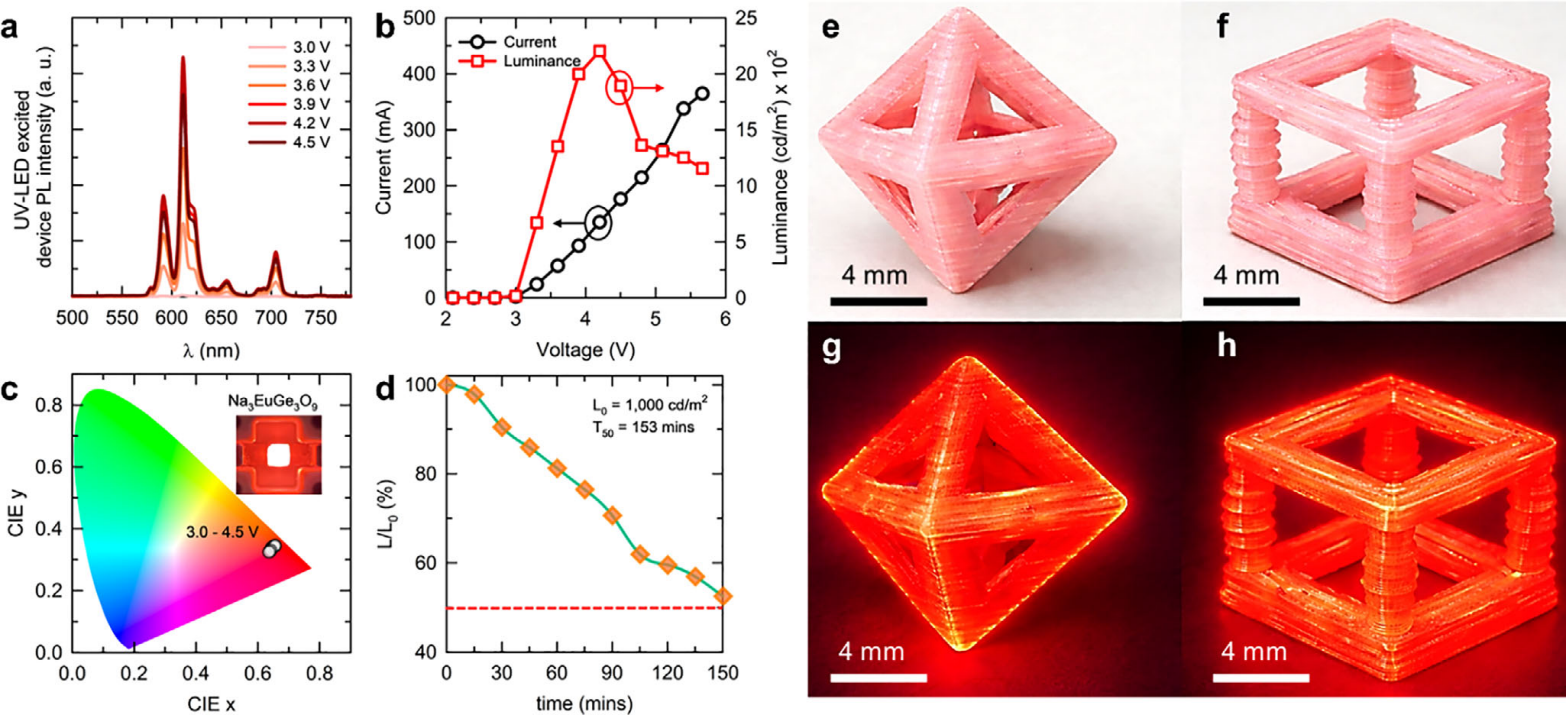
Device performance and additive‐manufactured color‐converting photonic structures based on NEGO. (a) EL spectra of the UV‐excited color‐converting device incorporating NEGO phosphor under forward bias voltages ranging from 3.0 to 4.5 V. (b) Current–voltage (*I*–*V*) characteristics and corresponding luminance of the fabricated color‐converting device. (c) CIE chromaticity coordinates of the device measured at different operating voltages, with the inset showing the fabricated color‐converting device under UV excitation. (d) Operational stability of the device, showing the normalized luminance (L/L_0_) as a function of continuous operating time (L_0_ = 1000 cd m^−2^; T_50_ = 153 min). (e,f) Optical photographs of representative three‐dimensional photonic architectures fabricated by extrusion‐based 3D printing using the NEGO‐based printable composite. (g,h) Corresponding photographs of the 3D‐printed architectures under UV illumination, exhibiting bright, uniform red emission throughout the printed structures. Scale bars: 4 mm.

Beyond demonstrating efficient and stable color conversion in optoelectronic devices, the integration of luminescent materials into complex three‐dimensional architectures is important for emerging photonic technologies, for which conventional ceramic phosphors are often limited by brittleness and poor processability. To explore this potential, NEGO was incorporated into a printable matrix to fabricate complex three‐dimensional architectures using extrusion‐based 3D printing (Figure 5e,f). The Ge‐containing composition exhibited excellent compatibility with the printable matrix, enabling the formation of mechanically robust lattice structures with high printing fidelity. Under UV illumination, the printed architectures exhibited intense, spatially uniform red luminescence throughout the structure without noticeable brightness variation (Figure 5g,h), indicating homogeneous phosphor dispersion and efficient light conversion. Thus, the EL device and 3D printing demonstrations highlight two complementary advantages of NEGO: its ability to deliver efficient, spectrally stable red color conversion under electrical operation and its compatibility with additive manufacturing for architecture photonic structures. These results establish NEGO as a versatile platform for next‐generation color conversion devices and additive‐manufactured photonic applications. Overall, these results validate NEGO as a promising red phosphor for high‐performance color‐conversion devices and next‐generation additive‐manufactured photonic applications.

## 3 Conclusions

In summary, this study establishes local activator stability as a key descriptor for understanding and designing thermally robust luminescent materials. Although NEGO and NESO share the same non‐centrosymmetric *P2_1_2_1_2_1_* structure, subtle changes in the local chemical environment induce substantial differences in dipole modulation, Eu─O bonding, and thermal quenching behavior. Judd–Ofelt calculations further reveal enhanced local symmetry breaking and Eu─O covalency in NEGO, confirming its higher electric dipole transition probability. More importantly, our combined experimental and theoretical analyses show that thermal robustness cannot be reliably inferred from a single bulk or static descriptor, such as the Debye temperature or the ground‐state bandgap. NESO is the globally stiffer host, whereas NEGO combines lower host phonon energies with stronger average Eu─O bonding and smaller displacement‐induced changes in the Eu‐centered structural and electronic descriptors. These results shift the emphasis from relying on bulk lattice descriptors alone to the combined evaluation of local bonding, host phonon energies, and displacement‐sensitive activator properties. The present calculations do not identify a unique dominant nonradiative pathway but provide a comparative framework for rationalizing the contrasting thermal‐quenching behaviors of NESO and NEGO. The demonstrated color‐conversion and 3D‐printed photonic applications further highlight the technological versatility of this material platform. Overall, this study provides a general framework for connecting local symmetry and chemical bonding with thermal quenching resistance, offering a pathway toward the rational design of high‐efficiency, thermally robust luminescent materials.

## Author Contributions

**Noolu. Srinivasa Manikanta Viswanath**: conceptualization, investigation, formal analysis, writing – original draft. **Jeong Hui Kim**: investigation, formal analysis. **Yeongjun Yoon**: writing – original draft, software, investigation. **Sung Woo Jang**: formal analysis, investigation. **Sanjith Unithrattil**: investigation, formal analysis. **San Ha Choi**: investigation, formal analysis. **Kyeounghak Kim**: software, supervision. **Won Bin Im**: supervision, conceptualization, writing – review and editing.

## Conflicts of Interest

The authors declare no conflicts of interest.

## Supporting information




**Supporting File**: advs78623‐sup‐0001‐SuppMat.docx

## Data Availability

The data that support the findings of this study are available on request from the corresponding author. The data are not publicly available due to privacy or ethical restrictions.
