# Supplementary material for: Beyond the Debye Temperature: A Unified Microscopic Framework for Designing Thermally Stable Red‐Emitting Phosphors
Source: Adv Sci (Weinh). 2026 Nov 9:e78623. Online ahead of print. doi: 10.1002/advs.78623 (PMC13749008; doi:10.1002/advs.78623)
Supplement: Supplementary file 1 [file ADVS-9999-e78623-s001.docx]

Supporting Information

Beyond the Debye Temperature: A Unified Microscopic Framework for Designing Thermally Stable Red-Emitting Phosphors

*Noolu. Srinivasa Manikanta Viswanath,^1$^ Jeong Hui Kim,^1$^ Yeongjun Yoon,^2$^ Sung Woo Jang,^1^ Sanjith Unithrattil,^1^ San Ha Choi, ^1^ Kyeounghak Kim,^2*^ and Won Bin Im^1*^*

Dr. N.S.M. Viswanath, JH Kim, SW Jang, Dr. Sanjith, and Prof. WB IM

^1^Division of Materials Science and Engineering, Hanyang University, 222 Wangsimni-ro, Seongdong-gu, Seoul, 04763, Republic of Korea
E-mail: imwonbin@hanyang.ac.kr

Y Yoon, and Prof. K Kim

^2^Division of Chemical Engineering, Hanyang University, 222 Wangsimni-ro, Seongdong-gu, Seoul, 04763, Republic of Korea

E-mail: [chemekim@hanyang.ac.kr](mailto:chemekim@hanyang.ac.kr)

^$^ These authors equally contributed to this work

**Experimental section**

**Chemicals**

High-purity sodium carbonate (Na_2_CO_3_, ≥99.5%), europium oxide (Eu_2_O_3_, 99.99%), germanium dioxide (GeO_2_, 99.999%), and silicon dioxide (SiO_2_, ≥99.9%) were purchased from Sigma-Aldrich and used as received without further purification.

**Synthesis of Na_3_EuSi_3_O_9_ (NESO)**

Polycrystalline Na₃EuSi₃O₉ (NESO) was synthesized by a conventional high-temperature solid-state reaction. Stoichiometric amounts of Na_2_CO_3_, Eu₂O₃, and SiO_2_ were weighed according to the stochiometric ratio and thoroughly mixed by grinding in an agate mortar using acetone as the milling medium until a homogeneous slurry was obtained. After drying, the powder mixture was transferred into an alumina crucible and calcined in air at 1100°C for 6 h. The furnace was then allowed to cool naturally to room temperature. The obtained product was reground to obtain a fine powder for subsequent structural and optical characterization.

**Synthesis of Na_3_EuGe_3_O_9_ (NEGO)**

Na₃EuGe₃O₉ (NEGO) phosphor was prepared using the same conventional solid-state reaction method. Stoichiometric quantities of Na_2_CO_3_, Eu₂O₃, and GeO_2_ were thoroughly ground in an agate mortar with acetone to ensure homogeneous mixing. The dried precursor powder was placed in an alumina crucible and fired in ambient air at 1100°C for 6 h. After natural cooling to room temperature, the sintered product was crushed and ground into a fine powder for further characterization.

**Structural and Morphology characterizations**

The structures of the as-synthesized samples were characterized by XRD (Miniflex 600) using a diffracted beam monochromator set for Cu-Kα radiation (λ = 1.54056 Å). The 2θ scan range was 10-70º with a step size of 0.01º. A three-dimensional visualization system for electronic and structural analysis (VESTA) was used to draw the crystal structures. SEM images were obtained using a NOVA NANO SEM-450 instrument.

**Optical characterizations**

Steady-state PL spectra of the samples were recorded using a Hitachi F-7000 fluorescence spectrophotometer. The variation in PL intensity during heating was measured by connecting a Hitachi F-7000 fluorescence to an integrated heater, temperature controller, and thermal sensor. A time-correlated single-photon counting system (TimeHarp-260, PicoQuant GmbH, Germany) was used to count the emitted photons. The PLQYs of the as-synthesized samples were measured using a HORIBA FluoroMax Plus spectrofluorometer. The absolute PLQY of the samples were measured using a HORIBA FluoroMax Plus spectrofluorometer paired with K-sphere (S/N: 11503820) petite internal, directly coupled with Integrating Sphere. The excitation source was standard 75 W xenon arc, attached to the front of the sample compartment. The electroluminescence spectra, current-voltage-luminance (I-V-L) characteristics, CIE color coordinates of the LED devices were measured using a spectroradiometer (CS-2000, Konica Minolta) with a Keithley 2400 source meter under ambient conditions. The electroluminescence spectra, current-voltage-luminance (I-V-L) characteristics, CIE color coordinates of the LED devices were measured using a spectroradiometer (CS-2000, Konica Minolta) with a Keithley 2400 source meter under ambient conditions.

**Temperature-dependent photoluminescence measurements**: TQ measurements were performed under 465 nm excitation for NESO and NEGO, corresponding to their respective maximum PLE intensities. For comparison, the commercial phosphors KSF:Mn^4+^ and CaAlSiN_3_:Eu^2+^ were excited at 450 nm, corresponding to their maximum PLE intensities. Each phosphor was measured under its respective optimal excitation wavelength to evaluate its temperature-dependent emission behavior.

**3D Printing Setup:** A custom-built stage-moving 3D printer (MutoLabs Co., Ltd., Republic of Korea) was used for fused deposition modeling (FDM). In this configuration, the print bed translated along the x- and y-axes, while the nozzle moved along the z-axis. 3D models were sliced into 2D layers using CURA (Ultimaker). The printer has FDM module: Filament module – for FDM printing of composite filaments prepared from thermoplastic resins and luminescent fillers.

**Fabricating of the Na_3_EuGe_3_O_9_ ink composite and color conversion device**

To synthesize Na_3_EuGe_3_O_9_ ink composite, the 0.5 g of Na_3_EuGe_3_O_9_ polycrystals were uniformly mixed with polydimethylsiloxane (PDMS, DOW Corning SYLGARD^TM^ 184 Silicone Elastomer Base) and curing agent (DOW Corning SYLGARD^TM^ 184 Silicone Elastomer Curing Agent) in a vial for 1 h. (Weight ratio of 1:1:0.1) After fully stirring the mixed composite, 0.1 ml of composite was directly coated onto the 395 nm UV LED chips and annealed at 100^o^C for 1 hours in an oven.

**Fabrication of Composite Filaments**

PLA and DCM were combined (1:9 w/w) and stirred for 30 min. After complete dissolution, Na_3_EuGe_3_O_9_ were added (PLA:powder = 3:1 by weight) and stirred for another 30 min. The mixture was injected into silicone tubing using a syringe. DCM was evaporated at room temperature, and the filament was extracted after 24 h.

**FDM Printing process**

Composite filaments were prepared by co-dissolving Na_3_EuGe_3_O_9_ and poly(lactic acid) (PLA) in dichloromethane (DCM), drying in silicone tubing, and spooling. The composite filament was loaded into the 3D printer, and the printing process was initiated using the designated printing program. Printing temperatures were 210°C for composite filaments. Stable extrusion was achieved up to 50 wt.% loading without nozzle clogging or mechanical failure.

**Computational Details**

All spin-polarized density functional theory (DFT) calculations were performed using the Vienna Ab-initio Simulation Package (VASP, version 6.5.1)^[1]^ on a high-performance computing (HPC) system (Equipment ID: NFEC-2025-02-303332). The interactions between valence electrons and ionic cores were modeled using projector augmented-wave (PAW) pseudopotentials with a plane-wave cutoff energy of 520 eV.^[2]^ The energy convergence threshold for the self-consistent field (SCF) cycles was set to 10^−6^ eV, and geometry optimizations were carried out until the maximum residual force on any atom was less than 0.02 eV/Å. The Brillouin zone was sampled using a Γ-centered grid with a k-point spacing of 0.03 2π/Å. Subsequent bonding analyses via Crystal Orbital Hamiltonian Populations (COHP) were conducted using the LOBSTER program.^[3]^ Data extraction for the electronic- and phonon- density of states (DOS), band structures, and thermodynamic properties was facilitated by the py4vasp library.^[4]^

To model the Na_3_EuSi_3_O_9_ and Na_3_EuGe_3_O_9_ crystals, we employed the restored regularized strongly constrained and appropriately normed (r^2^SCAN) functional. While r^2^SCAN delivers robust numerical accuracy and computational efficiency,^[5]^ these crystals are complex oxide systems containing strongly correlated Eu 4f electrons. In such highly correlated systems, standard semi-local and meta-GGA functionals can still exhibit significant electronic self-interaction errors. To mitigate these shortcomings and accurately capture the highly localized nature of the Eu 4f states, an on-site Hubbard U correction was incorporated, establishing the r^2^SCAN+U framework. By screening against the experimental optical bandgap of Na_3_EuSi_3_O_9_ (3.40 eV), an effective U (U_eff_) value of 6.0 eV was chosen (**Table S5**).

To account for electron–phonon interactions at finite temperatures and the quantum zero-point renormalization of the bandgap, we implemented the special displacement method proposed by Zacharias and Giustino (ZG).^[6]^ The ZG configuration effectively captures electron–phonon coupling by introducing a specific set of atomic displacements that reproduce the thermodynamic disorder of the nuclear positions. Given the substantial computational cost associated with the large 256-atom unit cells of Na_3_EuSi_3_O_9_ and Na_3_EuGe_3_O_9_, we performed the ZG calculations utilizing 1×1×1 supercells. These one-shot ZG configurations were generated at temperatures of 0, 85, 110, 135, 160, 185, 210, 235, 260, 300, 350, 400, and 450 K.

To theoretically investigate the non-radiative decay process and thermal quenching behavior in Na_3_EuSi_3_O_9_ and Na_3_EuGe_3_O_9_ hosts, we employed a multiphonon relaxation model based on the harmonic oscillator approximation. The fundamental phonon frequency (ν) of the host lattice is governed by the bond strength (*k*) and the reduced mass (μ) of the vibrating atoms, expressed as:

$\nu=\frac{1}{2\pi}\sqrt{\frac{k}{\mu}}$	eqn. (1)

$\mu=m_{1}\parallel m_{2}=\left( m_{1}m_{2} \right)/\left( m_{1}+m_{2} \right)$	eqn. (2)

where m_1_ and m_2_ represent the atomic masses of the constituent atoms forming the specific bond. Applying this diatomic harmonic oscillator model, the effective reduced masses (μ) for the representative Si-O and Ge-O bonds are calculated to be 10.193 amu and 13.111 amu, respectively. Given that Ge-O bonds are generally weaker than the Si-O bonds (*k*_Ge-O_ < *k*_Si-O_) (**Table S6**), the Si-based system inherently possesses a higher maximum phonon frequency (ℏω_max_).

The probability of non-radiative transition via multiphonon emission is determined by the number of phonons (*p*) required to bridge the energy gap (∆E_mp_ $\approx$ 12,000 cm^-1^).^[7]^ The order of the multiphonon process (*p*) is defined as:

$p=\frac{\Delta E_{\text{mp}}}{\hbar\omega_{\text{max}}}$	eqn. (3)

The required number of phonon processes is estimated to be 10.8 for the Na_3_EuSi_3_O_9_ (ℏω_max_ = 1,112 cm^−1^) and Na_3_EuGe_3_O_9_ for the Ge system (ℏω_max_ = 896 cm^−1^).

Finally, according to the energy gap law, the non-radiative decay rate (W_nr_) exhibits an exponential dependence on the number of required phonons (*p*):

$W_{\text{nr}}=A ⅇ^{-\alpha p}$	eqn. (4)

where A and α are constants specific to the host environment.

**Refractive-Index Values and Judd–Ofelt Analysis Procedure**

The Judd–Ofelt (J–O) parameters and related radiative properties of Na₃EuSi₃O₉ (NESO) and Na₃EuGe₃O₉ (NEGO) were calculated using the JOES (Judd–Ofelt from Emission Spectrum) software based on the experimentally measured Eu³⁺ photoluminescence emission spectra. The refractive-index values for NESO and NEGO were approximated using the corresponding values of chemically related reference materials, Eu₂SiO₅ and Eu₂GeO₅, respectively, obtained from the RefractiveIndex.INFO database (<https://refractiveindex.info/>), where suitable wavelength-dependent data were available. These values were used as approximations rather than as experimentally measured refractive indices of the investigated phosphors.

**Procedure for Judd–Ofelt Analysis Using JOES Software**

1. **Preparation of emission spectra:** The experimentally measured photoluminescence emission spectra of NESO and NEGO were exported from the spectrofluorometer as wavelength–intensity data in a compatible file format.
2. **Importing spectral data:** The emission data were imported into the JOES software, following the instructions provided in the software tutorial.
3. **Identification of emission transitions:** The characteristic Eu³⁺ emission bands corresponding to the transitions from the ⁵D₀ excited state to the ⁷F₁, ⁷F₂, ⁷F₄, and other resolvable ⁷Fⱼ levels were identified. The integrated intensities of the relevant emission bands were determined according to the software's requirements.
4. **Refractive-index input:** The wavelength-dependent refractive-index values of Eu₂SiO₅ and Eu₂GeO₅ were obtained from the RefractiveIndex.INFO database and used as approximate inputs for NESO and NEGO, respectively. Where required, the values corresponding to the relevant emission wavelengths were selected in accordance with the software's input requirements.
5. **Calculation of J–O parameters:** The emission spectral information and required optical inputs were processed using JOES to calculate the applicable J–O intensity parameters (Ω₂, Ω₄, and Ω₆, where supported), radiative transition probabilities, and branching ratios.
6. **Comparative analysis:** The same analysis procedure was applied to both NESO and NEGO to compare their calculated radiative properties and investigate changes in the local Eu³⁺ coordination environment associated with Ge substitution.

The calculated J–O parameters and related radiative properties are derived quantities rather than direct experimental measurements. Since approximate refractive-index values from chemically related reference materials were used, this assumption should be considered when interpreting the results. The increase in Ω₂ and the asymmetry ratio may indicate changes in the local Eu³⁺ environment and the electric-dipole character of the emission transitions, but these parameters alone do not directly establish the magnitude of the local dipole moment or Eu–O covalency.

**Figure S1.** Spin-polarized electronic band structures of NESO showing the spin-up (Si-Up) and spin-down (Si-Down) electronic states along the high-symmetry paths of the Brillouin zone. The bands reveal the electronic structure and spin-dependent dispersion of the material, with the Eu-derived states located near the band edges.

**Figure S2.** Spin-polarized electronic band structures of NEGO showing the spin-up (Ge-Up) and spin-down (Ge-Down) electronic states along the high-symmetry paths of the Brillouin zone. The bands reveal the electronic structure and spin-dependent dispersion of the material, with the Eu-derived states located near the band edges.

**Figure S3.** Tauc plots derived from diffuse reflectance spectra using the Kubelka–Munk function, F(R) for NESO and NEGO. The optical bandgaps were estimated by extrapolating the linear absorption regions, yielding bandgap energies of 3.4 eV for NESO and 3.1 eV for NEGO, respectively.

**Figure S4.** Scanning electron microscopy (SEM) images of (a) NESO and (b) NEGO powders. Both samples exhibit irregularly shaped, fractured particles with rough surfaces. Compared with NESO, NEGO displays relatively larger, more angular flakes with smoother cleavage planes, indicating differences in particle morphology after processing.

**Figure S5.** SEM micrograph and EDS elemental mapping of NESO. The SEM image shows the morphology of a representative NEGO particle. Corresponding energy-dispersive X-ray spectroscopy (EDS) elemental maps reveal the spatial distribution of Na, Eu, Si, and O, indicating that the constituent elements are homogeneously distributed throughout the particle. The SEM image has a scale bar of 50 µm.

**Figure S6.** SEM micrograph and EDS elemental mapping of NEGO. The SEM image shows the morphology of a representative NEGO particle. Corresponding energy-dispersive X-ray spectroscopy (EDS) elemental maps reveal the spatial distribution of Na, Eu, Si, and O, indicating that the constituent elements are homogeneously distributed throughout the particle. The SEM image has a scale bar of 100 µm.

**Figure S7**. Thermal stability and cycling reliability of NEGO. (a) Temperature-dependent relative PL intensity during heating and cooling, showing approximately 92% intensity retention at 473 K. (b) Reproducibility of the PL response over six heating–cooling cycles between 298 and 473 K. (c–e) PL spectra recorded at 298 and 473 K during the first heating cycle. (f–h) PL spectra recorded during the first, second, and third cooling cycles, demonstrating the preservation of emission profiles and peak positions. These results confirm the excellent thermal robustness, reversibility, and short-term cycling stability of NEGO.

**Figure S8**. Arrhenius plots for NESO and NEGO, used to evaluate the activation energy barriers associated with thermal quenching. The linear fitting results indicate the enhanced resistance of NEGO to thermal quenching compared with NESO.

**Figure S9.** PL spectra of (a) commercial KSF:Mn^4+^ phosphor and (b) NEGO phosphor after exposure to water for 0, 1, and 3 days. The KSF:Mn^4+^ phosphor exhibits a significant decrease in emission intensity with increasing immersion time, indicating poor water stability. In contrast, the NEGO phosphor retains nearly unchanged PL intensity after 3 days of water exposure, demonstrating its superior moisture resistance and excellent optical stability.

**Figure S10**. Structural, morphological, and optical characterization of four independently synthesized samples before and after water immersion: (a) XRD patterns, (b) SEM images, and (c) PL spectra after three days of water immersion, demonstrating structural preservation, morphological integrity, and reproducible luminescence performance.

**Figure S11.** Comparison of the PL emission spectra of the NEGO phosphor under conventional optical excitation and UV-LED device excitation. The normalized PL spectra obtained from the UV-LED-excited device closely match those measured under conventional PL excitation, indicating that the NEGO phosphor maintains its characteristic emission profile after device integration and exhibits excellent spectral stability.

**Table S1** Lattice parameters of Na_3_EuGe_3_O_9_ obtained from Rietveld refinement of X-ray diffraction profile. The numbers in parentheses are the estimated standard deviations of the least significant figure.

| sample | Na_3_EuGe_3_O_9_ | Na_3_EuSi_3_O_9_ |
| --- | --- | --- |
| radiation type | X-ray | X-ray |
| 2θ range (degree) | 10-100 | 10-100 |
| *T*/*K* | 295 | 295 |
| symmetry | orthorhombic | orthorhombic |
| space group | *P 2_1_ 2_1_ 2_1_* | *P 2_1_ 2_1_ 2_1_* |
| *Z* | 16 | 16 |
| *a*/Å | 15.4905(4) | 15.4895(7) |
| *b*/Å | 15.6680(4) | 15.6675(5) |
| *c*/Å | 15.7682(4) | 15.7672(3) |
| *V*/Å^3^ | 3827.03(12) | 3826.41(5) |
| *R_wp_* | 4.35 % | 4.78% |
| *R_p_* | 2.21 % | 2.28% |
| *Χ^2^* | 4.89 | 4.95 |

**Table S2** Refined structural parameters of Na_3_EuSi_3_O_9_ obtained from the X-ray powder diffraction data at room temperature. The numbers in parentheses are the estimated standard deviations of the least significant figure.

| atom | site | *x* | *y* | *z* | g | 100×U_iso_/Å |
| --- | --- | --- | --- | --- | --- | --- |
| Na1 | *4a* | 0.1464(7) | 0.3876(7) | 0.4015(8) | 1 | 0.025(7) |
| Na2 | *4a* | 0.1021(7) | 0.1434(7) | 0.6166(8) | 1 | 0.051(7) |
| Na3 | *4a* | 0.3868(6) | 0.0987(7) | 0.6385(7) | 1 | 0.002(6) |
| Na4 | *4a* | 0.4386(8) | 0.4361(8) | 1.0185(8) | 1 | 0.066(8) |
| Na5 | *4a* | 0.1447(7) | 0.1436(8) | 0.8965(7) | 1 | 0.002(2) |
| Na6 | *4a* | 0.1023(7) | 0.3975(6) | 0.8479(6) | 1 | 0.041(3) |
| Na7 | *4a* | 0.1841(8) | 0.1491(9) | 0.3515(8) | 1 | 0.067(5) |
| Na8 | *4a* | 0.0188(9) | 0.0294(9) | 0.4903(9) | 1 | 0.002(8) |
| Na9 | *4a* | 0.0951(9) | 0.3381(8) | 0.1184(9) | 1 | 0.002(1) |
| Na10 | *4a* | 0.2855(8) | 0.2443(8) | 0.2186(9) | 1 | 0.048(8) |
| Na11 | *4a* | 0.4112(9) | 0.1131(9) | 0.3964(9) | 1 | 0.082(5) |
| Na12 | *4a* | 0.2639(9) | 0.2818(9) | 0.7690(9) | 1 | 0.035(3) |
| Eu1 | *4a* | 0.3817(2) | 0.3599(2) | 0.3775(2) | 1 | 0.001(4) |
| Eu2 | *4a* | 0.1247(2) | 0.1292(2) | 0.1163(2) | 1 | 0.002(4) |
| Eu3 | *4a* | 0.3696(2) | 0.1172(2) | 0.8670(2) | 1 | 0.043(4) |
| Eu4 | *4a* | 0.1395(2) | 0.3674(2) | 0.6109(2) | 1 | 0.031(4) |
| Si1 | *4a* | 0.4748(5) | 0.4574(5) | 0.7353(5) | 1 | 0.002(1) |
| Si2 | *4a* | 0.4916(5) | 0.2427(5) | 0.0298(5) | 1 | 0.059(1) |
| Si3 | *4a* | 0.2178(5) | 0.4936(4) | 0.2434(4) | 1 | 0.043(1) |
| Si4 | *4a* | 0.0217(4) | 0.0103(4) | 0.7553(4) | 1 | 0.023(1) |
| Si5 | *4a* | 0.5108(5) | 0.2611(5) | 0.7215(4) | 1 | 0.002(1) |
| Si6 | *4a* | 0.2035(5) | 0.4534(4) | 0.1975(5) | 1 | 0.051(1) |
| Si7 | *4a* | 0.2653(5) | 0.0344(5) | 0.2335(5) | 1 | 0.086(1) |
| Si8 | *4a* | 0.2706(5) | 0.2877(5) | 0.9964(5) | 1 | 0.002(1) |
| Si9 | *4a* | 0.4955(5) | 0.2534(5) | 0.5221(5) | 1 | 0.004(1) |
| Si10 | *4a* | 0.2416(5) | 0.0205(5) | 0.4735(5) | 1 | 0.002(1) |
| Si11 | *4a* | 0.0101(5) | 0.2464(5) | 0.7702(5) | 1 | 0.037(1) |
| Si12 | *4a* | 0.2824(6) | 0.2414(5) | 0.4976(5) | 1 | 0.028(1) |
| O1 | *4a* | 0.3116(1) | 0.4820(1) | 0.4848(1) | 1 | 0.047(7) |
| O2 | *4a* | 0.0959(1) | 0.2832(1) | 0.6920(1) | 1 | 0.008(3) |
| O3 | *4a* | 0.4144(1) | 0.2805(1) | 0.2792(1) | 1 | 0.002(5) |
| O4 | *4a* | 0.4804(1) | 0.1532 | 0.9877(1) | 1 | 0.002(5) |
| O5 | *4a* | 0.3829(1) | 0.2060(1) | 0.5797(1) | 1 | 0.062(3) |
| O6 | *4a* | 0.2834(1) | 0.0701(1) | 0.8098(1) | 1 | 0.013(2) |
| O7 | *4a* | 0.0227(0) | 0.3155(1) | 0.5260(7) | 1 | 0.0946 |
| O8 | *4a* | 0.0092(2) | 0.2738(7) | 0.8399(7) | 1 | 0.0176 |
| O9 | *4a* | 0.0480(3) | 0.4798(4) | 0.8692(4) | 1 | 0.0846 |
| O10 | *4a* | 0.0100(6) | 0.8276(7) | 0.4805(3) | 1 | 0.0548 |
| O11 | *4a* | 0.0317(5) | 0.9711(5) | 0.3316(1) | 1 | 0.0179 |
| O12 | *4a* | 0.0645(9) | 0.0950(5) | 0.7930(3) | 1 | 0.0175 |
| O13 | *4a* | 0.0323(5) | 0.7292(6) | 0.7353(0) | 1 | 0.0139 |
| O14 | *4a* | 0.0847(0) | 0.2670(5) | 0.2401(0) | 1 | 0.0474 |
| O15 | *4a* | 0.1035(6) | 0.7977(8) | 0.2702(5) | 1 | 0.0135 |
| O16 | *4a* | 0.1223(3) | 0.2770(3) | 0.7363(0) | 1 | 0.0165 |
| O17 | *4a* | 0.1287(8) | 0.9443(8) | 0.7532(8) | 1 | 0.0105 |
| O18 | *4a* | 0.1069(1) | 0.7225(3) | 0.5787(9) | 1 | 0.1219 |
| O19 | *4a* | 0.2207(1) | 0.1300(1) | 0.4906(1) | 1.00 | 0.010(1) |
| O20 | *4a* | 0.0028(1) | 0.2851(1) | 0.8404(1) | 1.00 | 0.002(5) |
| O21 | *4a* | 0.4492(1) | 0.3513(1) | 0.7063(1) | 1.00 | 0.002(9) |
| O22 | *4a* | 0.4548(1) | 0.3356(1) | 0.4720(1) | 1.00 | 0.003(1) |
| O23 | *4a* | 0.1985(1) | 0.4474(1) | 0.5157(1) | 1.00 | 0.002(5) |
| O24 | *4a* | 0.0279(1) | 0.0364(1) | 0.1399(1) | 1.00 | 0.025(1) |
| O25 | *4a* | 0.1840(1) | 0.0347(1) | 0.0297(1) | 1.00 | 0.001(3) |
| O26 | *4a* | 0.0887(1) | 0.1011(1) | 0.7658(1) | 1.00 | 0.044(7) |
| O27 | *4a* | 0.2345(1) | 0.0887(1) | 0.2136(1) | 1.00 | 0.008(9) |
| O28 | *4a* | 0.1271(1) | 0.4646(1) | 0.3309(1) | 1.00 | 0.078(7) |
| O29 | *4a* | 0.0239(1) | 0.5134(1) | 0.6140(1) | 1.00 | 0.078(5) |
| O30 | *4a* | 0.4343(1) | 0.2166(1) | 0.7623(1) | 1.00 | 0.019(2) |
| O31 | *4a* | 0.0343(1) | 0.1778(1) | 1.0316(1) | 1.00 | 0.016(7) |
| O32 | *4a* | 0.2118(1) | 0.2452(1) | 0.0433(1) | 1.00 | 0.186(5) |
| O33 | *4a* | 0.2869(1) | 0.3879(1) | 0.2726(1) | 1.00 | 0.025(3) |
| O34 | *4a* | 0.2740(1) | 0.2771(1) | 0.4266(1) | 1.00 | 0.025(3) |
| O35 | *4a* | 0.3346(1) | 0.0088(2) | 0.9867(1) | 1.00 | 0.033(5) |
| O36 | *4a* | 0.2300(1) | 0.2724(1) | 0.6001(1) | 1.00 | 0.025(7) |

**Table S3** Refined structural parameters of Na_3_EuGe_3_O_9_ obtained from the X-ray powder diffraction data at room temperature. The numbers in parentheses are the estimated standard deviations of the least significant figure.

| atom | site | *x* | *y* | *z* | g | 100×U_iso_/Å |
| --- | --- | --- | --- | --- | --- | --- |
| Na1 | *4a* | 0.0280(9) | 0.4832(4) | 0.4691(1) | 1 | 0.0179 |
| Na2 | *4a* | 0.0369(0) | 0.9961(5) | 0.5312(6) | 1 | 0.1555 |
| Na3 | *4a* | 0.1207(9) | 0.8476(3) | 0.8251(5) | 1 | 0.0157 |
| Na4 | *4a* | 0.0381(4) | 0.4519(6) | 0.0677(0) | 1 | 0.0948 |
| Na5 | *4a* | 0.1497(2) | 0.6489(1) | 0.3323(9) | 1 | 0.0179 |
| Na6 | *4a* | 0.1122(2) | 0.5855(6) | 0.6221(9) | 1 | 0.0002 |
| Na7 | *4a* | 0.1217(8) | 0.0926(6) | 0.3487(9) | 1 | 0.0128 |
| Na8 | *4a* | 0.1274(4) | 0.4141(6) | 0.8623(8) | 1 | 0.0189 |
| Na9 | *4a* | 0.1332(8) | 0.6072(6) | 0.1197(7) | 1 | 0.0168 |
| Na10 | *4a* | 0.1479(1) | 0.1270(5) | 0.9110(1) | 1 | 0.0187 |
| Na11 | *4a* | 0.2448(6) | 0.2589(7) | 0.2458(6) | 1 | 0.0319 |
| Na12 | *4a* | 0.2080(5) | 0.7873(2) | 0.2073(2) | 1 | 0.0129 |
| Eu1 | *4a* | 0.1044(7) | 0.1246(9) | 0.1207(9) | 1 | 0.0197 |
| Eu2 | *4a* | 0.1105(6) | 0.6234(4) | 0.8788(3) | 1 | 0.0198 |
| Eu3 | *4a* | 0.1219(4) | 0.3642(3) | 0.6160(1) | 1 | 0.0175 |
| Eu4 | *4a* | 0.1332(8) | 0.8784(9) | 0.3823(1) | 1 | 0.0112 |
| Ge1 | *4a* | 0.0214(6) | 0.7587(6) | 0.2046(3) | 1 | 0.0359 |
| Ge2 | *4a* | 0.0362(9) | 0.4911(0) | 0.2528(4) | 1 | 0.0156 |
| Ge3 | *4a* | 0.0140(5) | 0.7642(7) | 0.5389(2) | 1 | 0.0199 |
| Ge4 | *4a* | 0.0350(3) | 0.2229(4) | 0.7792(9) | 1 | 0.0228 |
| Ge5 | *4a* | 0.0281(1) | 0.0153(4) | 0.7731(9) | 1 | 0.0160 |
| Ge6 | *4a* | 0.0303(0) | 0.2225(0) | 0.4675(4) | 1 | 0.0101 |
| Ge7 | *4a* | 0.2281(6) | 0.9971(6) | 0.7339(5) | 1 | 0.0185 |
| Ge8 | *4a* | 0.2240(1) | 0.7249(3) | 0.5344(6) | 1 | 0.0179 |
| Ge9 | *4a* | 0.2254(4) | 0.4959(4) | 0.2576(6) | 1 | 0.0251 |
| Ge10 | *4a* | 0.2342(2) | 0.5172(7) | 0.4728(8) | 1 | 0.0451 |
| Ge11 | *4a* | 0.2436(0) | 0.2222(5) | 0.4751(2) | 1 | 0.0249 |
| Ge12 | *4a* | 0.2488(6) | 0.9783(9) | 0.0288(0) | 1 | 0.0335 |
| O1 | *4a* | 0.0568(9) | 0.5278(5) | 0.3413(7) | 1 | 0.5692 |
| O2 | *4a* | -0.0328(7) | 0.1737(8) | -0.0021(3) | 1 | 0.0106 |
| O3 | *4a* | -0.0449(0) | 0.4321(8) | 0.3535(3) | 1 | 0.1211 |
| O4 | *4a* | -0.0080(2) | 0.6320(6) | 0.9562(9) | 1 | 0.0175 |
| O5 | *4a* | 0.0403(7) | 0.7039(8) | 0.1722(0) | 1 | 0.1620 |
| O6 | *4a* | -0.0092(8) | 0.4711(7) | 0.6603(2) | 1 | 0.0155 |
| O7 | *4a* | 0.0227(0) | 0.3155(1) | 0.5260(7) | 1 | 0.0946 |
| O8 | *4a* | 0.0092(2) | 0.2738(7) | 0.8399(7) | 1 | 0.0176 |
| O9 | *4a* | 0.0480(3) | 0.4798(4) | 0.8692(4) | 1 | 0.0846 |
| O10 | *4a* | 0.0100(6) | 0.8276(7) | 0.4805(3) | 1 | 0.0548 |
| O11 | *4a* | 0.0317(5) | 0.9711(5) | 0.3316(1) | 1 | 0.0179 |
| O12 | *4a* | 0.0645(9) | 0.0950(5) | 0.7930(3) | 1 | 0.0175 |
| O13 | *4a* | 0.0323(5) | 0.7292(6) | 0.7353(0) | 1 | 0.0139 |
| O14 | *4a* | 0.0847(0) | 0.2670(5) | 0.2401(0) | 1 | 0.0474 |
| O15 | *4a* | 0.1035(6) | 0.7977(8) | 0.2702(5) | 1 | 0.0135 |
| O16 | *4a* | 0.1223(3) | 0.2770(3) | 0.7363(0) | 1 | 0.0165 |
| O17 | *4a* | 0.1287(8) | 0.9443(8) | 0.7532(8) | 1 | 0.0105 |
| O18 | *4a* | 0.1069(1) | 0.7225(3) | 0.5787(9) | 1 | 0.1219 |
| O19 | *4a* | 0.1289(1) | 0.4807(1) | 0.2189(3) | 1 | 0.0186 |
| O20 | *4a* | 0.0697(6) | 0.2350(7) | 0.4367(1) | 1 | 0.0256 |
| O21 | *4a* | 0.1408(9) | 0.4815(6) | 0.5040(0) | 1 | 0.0156 |
| O22 | *4a* | 0.1524(3) | 0.0326(3) | 0.0316(0) | 1 | 0.0116 |
| O23 | *4a* | 0.1948(7) | 0.5352(0) | 0.9486(8) | 1 | 0.0129 |
| O24 | *4a* | 0.1696(9) | 0.9727(8) | 0.4700(6) | 1 | 0.0451 |
| O25 | *4a* | 0.2134(4) | 0.2458(8) | 0.0833(5) | 1 | 0.0109 |
| O26 | *4a* | 0.2093(6) | 0.0876(2) | 0.2278(3) | 1 | 0.0104 |
| O27 | *4a* | 0.2130(5) | 0.7264(5) | 0.8937(1) | 1 | 0.0134 |
| O28 | *4a* | 0.1983(4) | 0.5487(4) | 0.3642(4) | 1 | 0.0184 |
| O29 | *4a* | 0.2513(1) | 0.6029(5) | 0.7921(5) | 1 | 0.0186 |
| O30 | *4a* | 0.1909(5) | 0.8473(4) | 0.9590(1) | 1 | 0.0184 |
| O31 | *4a* | 0.2295(1) | 0.0618(2) | 0.6077(3) | 1 | 0.3425 |
| O32 | *4a* | 0.2231(7) | 0.7691(2) | 0.4265(6) | 1 | 0.0019 |
| O33 | *4a* | 0.2262(1) | 0.4295(5) | 0.7219(8) | 1 | 0.0180 |
| O34 | *4a* | 0.2524(6) | 0.2894(2) | 0.5608(5) | 1 | 0.0149 |
| O35 | *4a* | 0.2397(3) | 0.9134(6) | 0.2857(6) | 1 | 0.0339 |
| O36 | *4a* | 0.2455(0) | 0.3833(6) | 0.0075(7) | 1 | 0.0156 |

**Table S4**. Fitted lifetime parameters and residuals for NESO and NEGO phosphors.

| Parameter | NESO | NEGO |
| --- | --- | --- |
| Amplitude A₁ | 0.2625 | 0.8016 |
| Lifetime τ₁ (ms) | 0.5002 | 0.9865 |
| Amplitude A₂ | 0.6628 | 0.0918 |
| Lifetime τ₂ (ms) | 1.1856 | 3.3443 |
| Background | 0.00178 | 0.00365 |
| Intensity-weighted average lifetime (ms) | 1.0625 | 1.5629 |
| R² | 0.99692 | 0.99652 |
| RMSE | 0.00740 | 0.00814 |
| Maximum absolute residual | 0.08766 | 0.10298 |
| Sum of squared residuals | 0.20875 | 0.25272 |

| U_eff_ (eV) | 4.0 | 4.5 | 5.0 | 5.5 | 6.0 |
| --- | --- | --- | --- | --- | --- |
| E_g, DFT_ (eV) | 2.53 | 2.74 | 2.95 | 3.17 | 3.39 |
| E_g, Exp._ (eV) | 3.40 | 3.40 | 3.40 | 3.40 | 3.40 |
| Error (eV) | 0.87 | 0.66 | 0.45 | 0.23 | 0.01 |

**Table S5.** Screening of the effective Hubbard U (U_eff_) parameter for the Eu 4f states in Na_3_EuSi_3_O_9_ within the r²SCAN+U framework. The table compares the DFT-calculated bandgaps (E_g, DFT_) against the experimental optical bandgap (E_g, Exp._ = 3.40 eV) across a range of applied U_eff_ values (4.0 to 6.0 eV).

**Table S6.** Calculated −integrated Crystal Orbital Hamiltonian Population (−ICOHP) values for the Eu-O, Si-O, and Ge-O bonds in the Na_3_EuSi_3_O_9_ and Na_3_EuGe_3_O_9_ systems.

| −ICOHP  (eV) | Na_3_EuSi_3_O_9_ | | Na_3_EuGe_3_O_9_ | |
| --- | --- | --- | --- | --- |
|  | Eu-O | Si-O | Eu-O | Ge-O |
| Minimum | 3.471 | 7.376 | 3.376 | 6.443 |
| Maximum | 4.250 | 9.138 | 4.384 | 8.499 |
| Averaged | 3.923 | 8.239 | 3.946 | 7.492 |

**Table S7.** Temperature-dependent structural distortion index (DI) of the EuO_6_ polyhedra in the Na_3_EuSi_3_O_9_ and Na_3_EuGe_3_O_9_ systems. The DI values represent the average calculated over the 16 distinct EuO_6_ polyhedra present in each structure. The "static lattice" refers to the idealized equilibrium geometry obtained directly from standard DFT optimization, where atoms are fixed at their minimum-energy positions without quantum zero-point motion or finite-temperature thermal effects.

| T (K) | DI of EuO_6_ | |
| --- | --- | --- |
|  | Na_3_EuSi_3_O_9_ | Na_3_EuGe_3_O_9_ |
| static lattice | 0.0098 | 0.0127 |
| 0 | 0.0224 | 0.0221 |
| 85 | 0.0226 | 0.0226 |
| 110 | 0.0229 | 0.0229 |
| 135 | 0.0233 | 0.0234 |
| 160 | 0.0237 | 0.0234 |
| 185 | 0.0242 | 0.0240 |
| 210 | 0.0248 | 0.0246 |
| 235 | 0.0254 | 0.0252 |
| 260 | 0.0260 | 0.0259 |
| 300 | 0.0272 | 0.0265 |
| 350 | 0.0282 | 0.0276 |
| 400 | 0.0296 | 0.0291 |
| 450 | 0.0309 | 0.0305 |

**References**

[1] G. Kresse, J. Furthmüller, Phys. Rev. B 1996, 54, 11169.

[2] G. Kresse, D. Joubert, Phys. Rev. B 1999, 59, 1758.

[3] R. Nelson, C. Ertural, J. George, V. L. Deringer, G. Hautier, R. Dronskowski, J. Comput. Chem. 2020, 41, 1931.

[4] VASP-dev, py4vasp (software), 2023–2025, https://github.com/vasp-dev/py4vasp.

[5] S. Swathilakshmi, R. Devi, G. S. Gautam, J. Chem. Theory Comput. 2023, 19, 4202.

[6] M. Zacharias, F. Giustino, Phys. Rev. B 2016, 94, 075125.

[7] W. T. Carnall, P. R. Fields, K. Rajnak, J. Chem. Phys. 1968, 49, 4450.
